# Histopathological and immunohistochemical characterization of *Burkholderia pseudomallei* lesions in an acute model of infection with BALB/c mice

**DOI:** 10.1111/iep.12258

**Published:** 2018-01-07

**Authors:** Waldo Luis García‐Jiménez, Francisco J. Salguero, Riccardo V. D'Elia

**Affiliations:** ^1^ Department of Pathology and Infectious Diseases School of Veterinary Medicine University of Surrey Guildford UK; ^2^ CBR Division Defence Science & Technology Laboratory Salisbury UK

**Keywords:** *Burkholderia pseudomallei*, immunohistochemistry, immunopathology, neutrophils

## Abstract

Organ tissue damage is a key contributor to host morbidity and mortality following infection with microbial agents. Severe immune responses, excessive cellular recruitment and necrosis of cells all play a role in disease pathology. Understanding the pathogenesis of disease can aid in identifying potential new therapeutic targets or simply act as a diagnostic tool. *Burkholderia pseudomallei* is a Gram‐negative bacterium that can cause acute and chronic diseases. The BALB/c mouse has been shown to be highly susceptible to aerosol challenge with *B. pseudomallei* and hence acts as a good model to study the acute and potentially lethal form of the disease melioidosis. In our study, BALB/c mice were challenged and culled at predetermined time points to generate a pathological time course of infection. Lung, liver and spleen were subjected to pathological and immunohistochemical analysis. The number and type of microscopic lesions within each organ, as well as the location and the mean percentage of neutrophils, B cells, T cells and *Burkholderia* capsule antigen within the lesions, were all characterized during the time course. Neutrophils were determined as the key player in tissue pathology and generation of lesions, with B cells playing an insignificant role. This detailed pathological assessment increases our understanding of *B. pseudomallei* disease.

*Burkholderia pseudomallei* is the causative agent of the disease melioidosis. It is an intracellular Gram‐negative bacterium that has the ability to infect numerous different cell types (David *et al*. 2015) and is naturally resistant to many antibiotics, due to efflux pumps (Mima & Schweizer 2010; Rhodes & Schweizer 2016).

Melioidosis is an emerging tropical disease, endemic to South‐East Asia and Northern Australia (Aldhous 2005; Kaestli *et al*. 2009; Valade *et al*. 2009). *Burkholderia pseudomallei* is known to cause diseases in humans via the aerosol route and via cutaneous lesions (Leelarasamee 1998). Depending on challenge dose and route of infection, it can either cause an acute disease where the immune response is inadequate and mortality rates are high or a chronic infection where the pathogen can reside for long times in localized regions (Wiersinga *et al*. 2006, 2009).

The natural ability of *B. pseudomallei* to infect via the aerosol route and its low challenge dose have also made this bacterium a potential biothreat agent of concern (Gilad *et al*. 2007). Currently, there is no licensed vaccine to prevent infection, and antibiotic treatment is suboptimal and often time‐consuming (Dance 2014). Single antibiotic dosing regimens are only partially effective, and combination treatments have to be given for prolonged periods, up to 6 months to clear the infection completely (Srinivasan *et al*. 2001). Therefore, there is a continued requirement to develop new therapies and thus a greater understanding of the disease.

Several animal models have been developed over the years to determine the host–pathogen interactions. The mouse model has been the most characterized with the BALB/c mouse being very susceptible to infection and the C57BL/6 mouse strain being more resistant (Barnes *et al*. 2001). This has led to the BALB/c mouse being mainly used for acute disease studies and the C57BL/6 strain for chronic models (Leakey *et al*. 1998; Hoppe *et al*. 1999; Liu *et al*. 2002; Barnes & Ketheesan 2005; Conejero *et al*. 2011; Bearss *et al*. 2017).

Despite growing research into this pathogen, there are still some gaps in the knowledge of the organ pathology and lesion development associated with the disease, although it has been documented that excessive immune response postinfection with *B. pseudomallei* is a significant contributor to host mortality (Ulett *et al*. 2000; Tan *et al*. 2008). Therefore, controlling tissue pathology and organ damage caused by an overactive immune response could represent an appropriate therapeutic strategy where an immunomodulatory drug may control the excessive inflammation and an antibiotic can control the bacterial spread (Laws *et al*. 2015). Preliminary histological studies have demonstrated in the BALB/c mouse that there are foci of acute inflammation and necrosis in the lung, liver and spleen following infection with *B. pseudomallei* BRI strain (Barnes *et al*. 2001; Lever *et al*. 2009). Additional histological studies have focused on the chronic C57BL/6 mouse model of infection with samples taken at day 20 and day 60 p.i, indicating that the spleen and liver are the most susceptible organs to bacterial growth and tissue destruction (Conejero *et al*. 2011).

In this study, we sought to carry out a detailed histopathological and immunohistochemical analysis using the BALB/c acute model and the *B. pseudomallei* K96243 human clinical isolate strain. Analyses were carried out at multiple time points during the short course of infection.

## Material and methods

### Experimental design

Forty male BALB/c mice (Charles River, UK), six to eight week old, were included in this study, split over two identical but independent experiments. Mice were randomly assigned into cages and transferred to a high‐containment Class III rigid isolator, where they were given unlimited access to food and water. Thirty mice were challenged with the *B. pseudomallei* strain K96243 by aerosol, using a Henderson‐type apparatus (Druett 1969) and a Collison nebulizer (May & Harper 1957), and ten were left as naïve controls. Bacteria were grown in Luria broth at 37°C on a rotary platform. Animals received a lethal dose ~ 20 ×  LD50 with an average challenge dose of 146 CFU. The challenge dose is the average calculated retained dose per mouse. Mice were sprayed for a total of 10 minutes with a 2‐minute subsamples (impinger) collected for bacterial enumeration. The impinger sample was serially diluted and plated in triplicates.

At 24 hpi, infected mice showed minor ruffling and hunching. At 48 hpi, ruffling and hunching were more pronounced and signs of conjunctivitis appeared. At 60 hpi, infected mice were either at or close to humane end point. Mice were culled at predetermined time points (TP1 = 24 hpi, TP2 = 48 hpi and TP3 = 60 hpi). Ten infected mice were culled at each time point while five naïve controls were culled at 24 hpi and five at 60 hpi.

### Histopathology

The lung, liver and spleen were all processed within less than 1 hour *post mortem* and immersed in neutral sodium salt‐buffered formalin. Samples were fixed for 1 month and embedded in paraffin wax, and 4‐μm sections were cut, dewaxed and rehydrated through xylene and alcohols to be finally washed in running tap water. Haematoxylin and eosin (H&E) stain was used for the histopathological analysis.

### Immunohistochemistry

The immunohistochemical detection of *B. pseudomallei* was performed with a murine monoclonal antibody (3VIE5) that reacts with a capsular polysaccharide of *B. pseudomallei*. For the characterization of cell composition of lesions, a rat monoclonal anti‐Ly‐6G (Invitrogen, Darmstadt, Germany), a rat monoclonal anti‐CD45R‐B220 (Invitrogen) and a rabbit polyclonal anti‐CD3 (Dako) were used as immunomarkers for neutrophils, B cells and T cells respectively.

Consecutive 4‐μm sections were dewaxed, rehydrated and placed in a fresh solution of 3% hydrogen peroxide in methanol for 15 minutes to block endogenous peroxidase activity. Samples were then washed for 10 minutes in tap water before being clipped into Shandon Sequenza clips (Thermo Scientific, Loughborough, Leistercershire, UK). After slides were mounted, epitope demasking was performed through enzymatic digestion, using a solution of 2% proteinase K (Dako) in Tris‐buffered saline (0.05 mol/L Tris–HCl pH 7.5 to 7.7). Then, the tissue sections were washed for 10 minutes with Tris‐buffered saline (TBS) (0.85% NaCl, 0.0605% Tris, adjusted to pH 7.5 using 1M HCl), and then, 190 μl of Universal Blocker™ Blocking Buffer in TBS (Thermo Scientific) was added to the slides as blocking agent. After twenty minutes, 190 μl of the primary antibody was added. The incubation time as well as the concentration varied for each primary antibody used (Table 1). The Vector M.O.M. Immunodetection kit (Vector, Peterborough, UK) was used for the anti‐*Burkholderia* antibody following the manufacturers’ indications. For anti‐Ly‐G6, anti‐CD45R‐B220 and anti‐CD3 antibodies, after two washes with TBS buffer, 190 μl of biotinylated link antibody and link block (Thermo Scientific) was added (Table 1), followed by two further buffer washes, 30 minutes later. Primary and secondary antibody binding was amplified using Ultra‐Sensitive ABC Peroxidase Rabbit IgG Staining Kit (Thermo Scientific) and visualized using the Vector^®^ *NovaRED™* Substrate Kit (Vector Laboratories, Burlingame, CA, USA). Unbound conjugate was removed prior to chromogen application with two buffer washes. Slides were then washed in purified water, removed from the coverplates and placed in a rack. Samples were rinsed with tap water for five minutes, before being placed in Mayer's Haematoxylin counterstain, followed by a further wash in tap water. Finally, sections were dehydrated, cleared and mounted for analysis.

**Table 1:** Antibodies and reagents used for immunohistochemistry

Primary antibody	Antibody type	Dilution	Supplier	Epitope demasking method	Link antibody (dilution)	Buffer
CD3	Rabbit vs Human CD3 (Polyclonal)	1/500	Dako (A0452)	Proteinase K (DAKO)	Goat vs Rabbit (1/1000)	TBS
CD45R‐B220	Rat vs Mouse CD45R‐B220 (Monoclonal)	1/100	Invitrogen (RA3‐6B2)	Proteinase K (DAKO)	Goat vs Rat (1/200)	TBS
Ly‐6G	Rat vs Mouse Ly‐6G (Monoclonal)	1/25	Invitrogen (RB6‐8C5)	Proteinase K (DAKO)	Goat vs Rat (1/200)	TBS
Anti‐Burkholderia	Mouse vs *Burkholderia pseudomallei* (Monoclonal)	1/50	DSTL Lever *et al*. (2009)	Proteinase K (DAKO)	Vector M.O.M. biotinylated mouse IgG (1/250)	TBS

### Image analysis and statistics

Immunolabelled sections were analysed using light microscopy and digital image analysis (Nikon NIS‐Br, Nikon, Japan).

The area of tissue with lesions was calculated using a full section of lung, liver and spleen. The slides were examined with the ×40 objective to give a final magnification of ×400, to ascertain the percentage of immunostained cells within the lesion.

To compare the mean number and type of lesions per time point, nonparametric Mann–Whitney's U‐test was used. Data were considered statistically significant when *P < *0.05.

For immunohistochemistry, the mean expression of anti‐CD3 antibody was compared for type of lesions and time points. Differences were considered significant at *P < *0.05. The results of immunohistochemical analysis are expressed as mean ± standard deviation (S.D).

Due to the staining characteristics or the localization of the immunolabelled cells, it was not possible to perform the image analysis for the antibodies anti‐*Burkholderia*, Ly‐6G and CD45B‐220. For this reason, the analysis of these antibodies has been made in a qualitative way.

All the statistical analyses were conducted using SPSS 19 software package (SPSS Inc., Chicago, IL, 60606, USA) and GraphPad Prism 4.0 (San Diego, CA, USA).

### Ethical approval

All procedures involving animals were conducted under project licences approved by internal ethical review and the Home Office, and in accordance with both the Animal (Scientific Procedures) Act (1986) and the 1989 Codes of Practice for the Housing and Care of Animals used in Scientific Procedures.

## Results

### Time course histopathology in the lung

**Figure 1:**
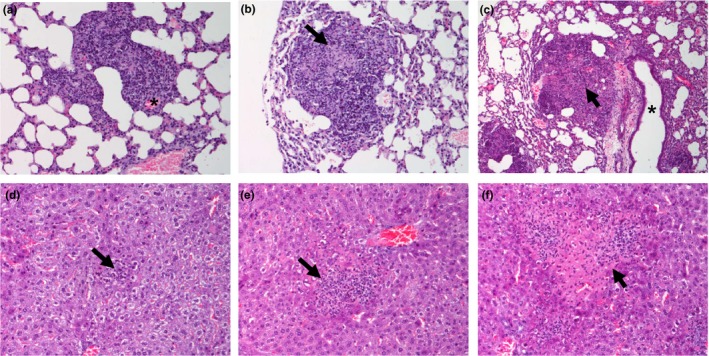
(a) H&E lung section from a type I lesion (200×). Inflammatory foci of macrophages, lymphocytes and some neutrophils close to a blood vessel (*). (b) H&E lung section from a type II lesion (100×). Foci of granulomatous pneumonia containing some epithelioid macrophages and neutrophils, some of which were necrotic (arrow). (c) H&E lung section from a type III lesion (100×). Large pyogranuloma adjacent to a bronchiole (*) formed by neutrophils with a necrotic centre (arrow) surrounded by few macrophages, lymphocytes and plasma cells. (*). (d) H&E liver section from a type I lesion (200×). Minimal foci of necrosis mainly due to infiltrated neutrophils (arrow). (e) H&E liver section from a type II lesion (200×). Moderate foci of necrosis formed by mixed inflammatory cells (arrow). (f) H&E liver section from a type III lesion (200×). Extensive foci of necrosis due to coalescent lesions (*). [Colour figure can be viewed at wileyonlinelibrary.com].

Lung lesions were classified into three categories according to cellular composition and evolution stage: type I, characterized by a discrete inflammatory foci of macrophages, lymphocytes and some neutrophils, usually close to blood vessels and/or bronchioles (Figure 1a); type II, defined as round lesions with areas of granulomatous pneumonia with similar distribution to those in type 1, but containing epithelioid macrophages and more neutrophils, some of which were necrotic (Figure 1b); and type III defined as large pyogranulomas mainly formed by neutrophils and with a necrotic centre surrounded by epithelioid macrophages, some lymphocytes and plasma cells (Figure 1c). The total number of lesions of each type was counted in a full lung section per animal.

**Figure 2:**
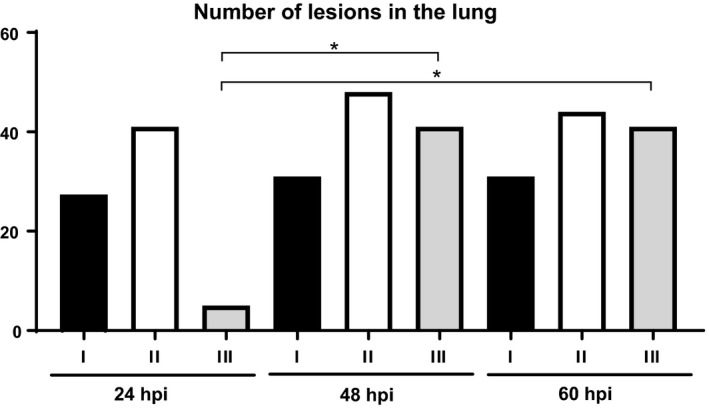
Time course histopathology in the lung. Total number of each type of lesions (I, II and III) observed per time point. At 48 and 60 hpi (TP2 and TP3), there was a statistically significant increase (*P *<* *0.05) in the total number of type III lesions (*) when compared to 24 hpi (TP1).

After 24 hpi, type II lesions were predominant in the lung followed by type I lesions. Type III lesions were exclusively observed in one animal at 24 hpi, while other animal did not show lesions at this time point. At 48 and 60 hpi, there was an increase in the total number of lesions, especially due to a statistically significant increase in type III lesions. Finally, at the last time point (60 hpi), the total number of lesions as well as the lesion types remained stable compared with 48 hpi (Figure 2).

### Time course histopathology in the liver

Lesions in the liver were classified, similar to lung, into three categories according to necrosis extension. Type I was characterized by a minimal foci of necrosis mainly due to infiltrated neutrophils (Figure 1d); type II was defined as moderate foci of necrosis formed by mixed inflammatory cells (Figure 1e); and type III was characterized by more extensive foci of necrosis frequently due to coalescent lesions (Figure 1f).

**Figure 3:**
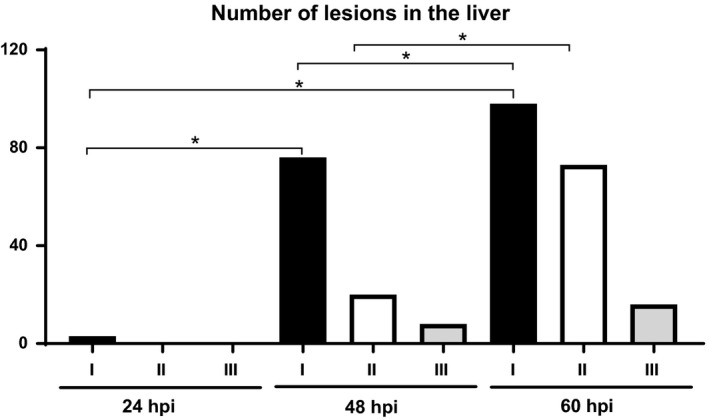
Time course histopathology in the liver. Total number of each type of lesions (I, II and III) observed per time point. An exponential increase in the number of lesions was observed at 48 hpi when compared to 24 hpi. At 60 hpi, there was an increase in the number of lesions when compared to 24 and 48 hpi, significantly higher for type I and II lesions (*P <* *0.05).

The total number of lesions of each category was counted in a full liver section per animal. Only two animals showed type I lesion in the liver at 24 hpi. However, there was an exponential increase in the number of lesions at 48 hpi, with type I lesions being the most observed. There was an increase in the number of all types of lesions at 60 hpi when compared to 24 and 48 hpi (Figure 3).

### Time course pathology in the spleen

The spleen showed a marked disruption of the normal tissue architecture. Lesions were typically pyogranulomatous, with abundant neutrophils and macrophages, mainly within the red pulp but also in the white pulp. No differentiation into types was performed for the splenic lesions. The percentage of tissue damaged in a full spleen section per animal was estimated to calculate the organ pathology score.

**Figure 4:**
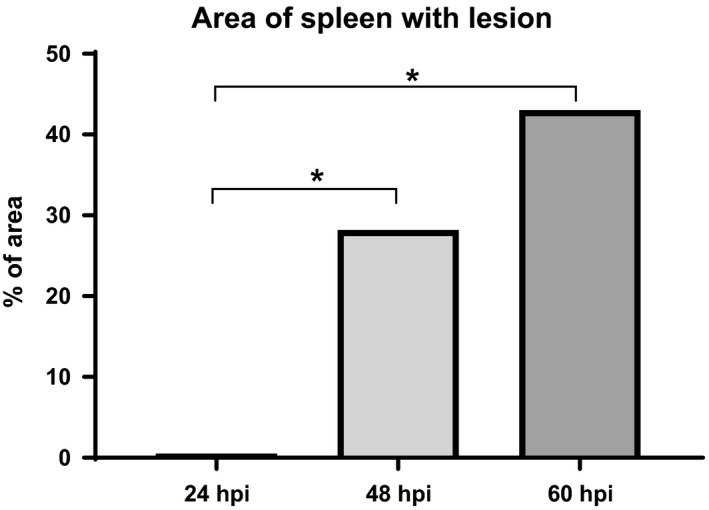
Time course pathology in the spleen. Percentage of spleen area affected per time point. A statistically significant (*P *<* *0.05) increase in the percentage of affected tissue at 48 and 60 hpi when compared to 24 hpi was observed (*).

Only one animal showed a single necrotic focus at 24 hpi. There was a statistically significant increase in the percentage of affected tissue at 48 hpi and 60 hpi when compared to 24 hpi with all animals showing lesions at this time point. At 60 hpi, also there was an increase in the percentage of affected tissue compared with 48 hpi, which was not statistically significant (Figure 4).

### Immunohistochemical analysis of *Burkholderia* spp. antigen and cell populations within lesions

#### Anti‐*Burkholderia* spp. antibody

**Figure 5:**
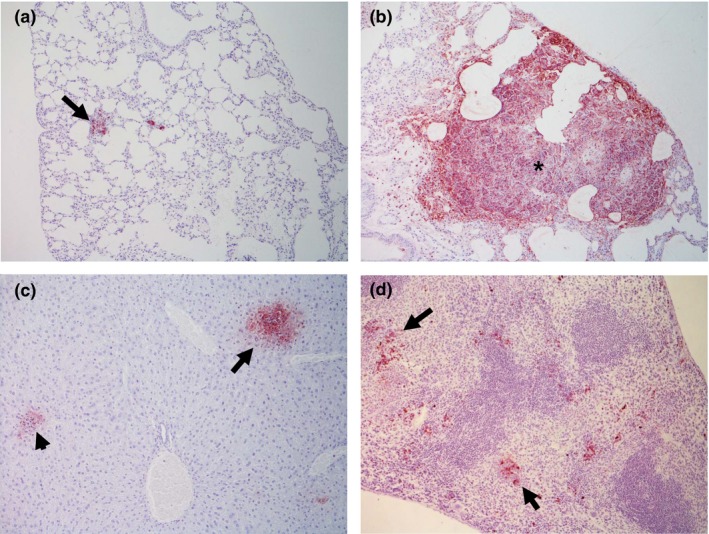
Immunohistochemically stained sections against a capsular polysaccharide of *Burkholderia pseudomallei*. (a) Type I lung lesions (arrow) at 24 hpi (100×) with positive reaction mainly within the cytoplasm of macrophages and neutrophils. (b) Extensive type III lesion at 60 hpi (100×) showing the antigen within the cytoplasm of macrophages and neutrophils associated with areas of consolidation and necrosis (*). (c) Positive immunohistochemical reaction within both degenerated and viable macrophages and neutrophils surrounding a focus of necrosis in a type II lesion (200×) in the liver (arrow) and a small type I lesion (arrowhead). (d) Positive immunohistochemical reaction within the spleen at 60 hpi, mostly affecting the red pulp (arrows) (100×). [Colour figure can be viewed at wileyonlinelibrary.com].

Immunohistochemistry against *Burkholderia* spp. capsular antigen showed extensive positive staining within lung, liver and spleen lesions. The positive reaction was located mainly within the cytoplasm of macrophages in type I lung lesions at 24 hpi (Figure 5a). However, staining in the lung became more extensive over time as infection progressed and antigen was located within the cytoplasm of both macrophages and neutrophils associated with areas of consolidation and necrosis in type II and III lesions (Figure 5b).

Positive reaction was also observed within both degenerated and viable macrophages and neutrophils surrounding the areas of necrosis in liver and spleen pyogranulomas (Figure 5c, d). No *Burkholderia* spp. capsular antigen was detected in uninfected controls.

#### Neutrophils (Ly‐6G)

**Figure 6:**
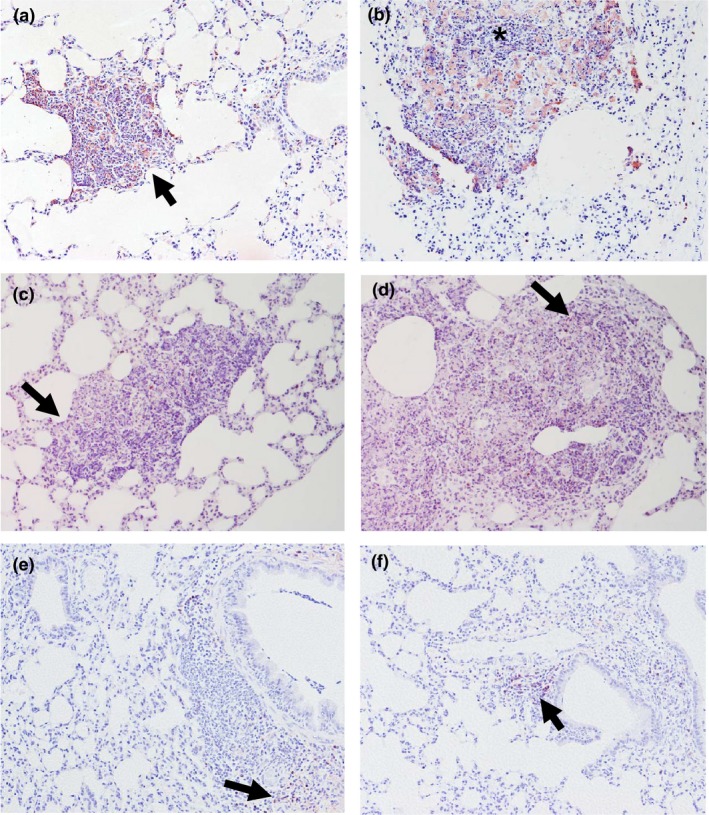
Immunohistochemical detection of Ly‐6G+ neutrophils (a, b), CD3 +  cells (c, d) and CD45R‐B220 cells (e, f). (a) Ly‐6G positive reaction in a type II lesion at 48 hpi (200×). Positive cells were mainly observed within the lesion but also in the adjacent tissue (arrow). (b) Ly‐6G+ cells in a type III lesion at 48 hpi (200×). High amount of Ly‐6G+ cells with a lighter staining in the centre of the lesion (*). (c) A low number of CD3 +  cells were observed in lung lesions. Scarce positive cells (arrow) in a type II lesion at 48 hpi (200×). Positive cells were mainly observed within the lesion but also in the adjacent tissue. (d) Very few CD3 +  cells at the periphery of a type III lesion (arrow) at 60 hpi (200×). (e) Immunohistochemical detection of very few B cells (CD45R‐B220 + ) within healthy tissue adjacent to a type II lesion (arrow) at 24 hpi (200×) (arrow). (f) ×Aggregate of B cells (arrow) forming a ‘nest’ close to a main bronchus and near to a type II lesion (200×) at 48 hpi. [Colour figure can be viewed at wileyonlinelibrary.com].

High numbers of Ly‐6G+ cells were detected in all lesion types in the lung. Positive cells were mainly observed within the lesion but also in the adjacent tissue (Figure 6a). Fewer positive cells were observed within the centre of the lesions, especially in the larger ones (Figure 6b). Fewer Ly‐6G+ cells were detected in hepatic and splenic lesions when compared to the lungs.

#### T lymphocytes (CD3)

**Figure 7:**
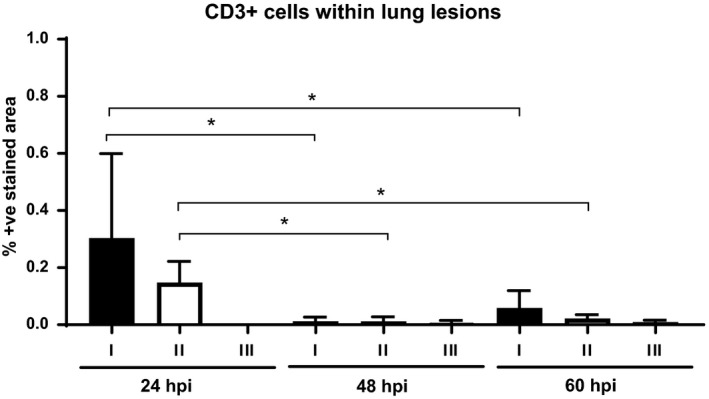
Immunohistochemical detection of CD3 +  cells within lung lesions as percentage of immunostained area. A statistically significant higher number of CD3 +  cells was observed in type I and II lesions at 24 hpi when compared to 48 and 60 hpi (*P <* *0.05).

Few CD3 +  cells were sparsely distributed within lesions independent of the lesion type. A small number of CD3 +  cells were observed in lung lesions (Figure 6c, d). However, a statistically significant higher number of CD3 +  cells were observed in type I and II lesions at 24 hpi when compared to 48 hpi and 60 hpi (Figure 7).

#### B lymphocytes (CD45R‐B220)

B lymphocytes do not seem to be involved in the development of *B. pseudomallei* lesions in our BALB/c model, as they were not observed in any type of lesions independently of the time point analysed. The number of B cells present in the adjacent healthy tissue was also small (Figure 6e), but occasionally they formed aggregates (Figure 6f). B cells were not observed in the hepatic lesions.

## Discussion

The organ pathology associated with *B. pseudomallei* infection is still not clearly understood. The BALB/c mouse model has been proposed as an appropriate model to evaluate the acute form of melioidosis in humans. However, the histopathology and immunopathology associated with this disease in BALB/c mice has been analysed infrequently (Barnes *et al*. 2001; Lever *et al*. 2009; Kager *et al*. 2014; Bearss *et al*. 2017).

Our study shows a detailed histopathological and immunohistochemical characterization of lung, liver and spleen pathologies using the BALB/c mice acute infection model and the *B. pseudomallei* K96243 human clinical isolate strain.

In our study, we found that 9/10 animals showed pyogranulomatous lesions within the lung as early as 24 hpi. These results are in line with previous studies demonstrating the high susceptibility of this mouse strain and how quickly the disease can evolve to the development of severe lesion types even at the first 24 hrs, as we found one animal showing type III lesions at this time point (TP1).

Previous studies described an exponential bacterial multiplication within the lung over the first 24 hpi, before the rate of replication became more gradual (Jeddeloh *et al*. 2003; Lever *et al*. 2009). This disease progression pattern is reflected within the microscopic lesions observed in our study, with an exponential increase in the number of lesions at 48 hpi (TP2), being later more stable at 60 hpi (TP3).

Similar patterns of initial exponential growth have been described in the liver and spleen once the bacteria are seeded from the lung. At this point, animals died because the damage in these organs became more severe while the rate of microorganism replication remained steady (Lever *et al*. 2009; Conejero *et al*. 2011).

In the study from Lever *et al*. (2009), no hepatic lesions were detected at 24 hpi and only two small foci of necrosis were shown at 48 hpi in the liver. In the same study, the lesions in the spleen described as multifocal acute necrotizing splenitis were not shown until 48 and 72 hpi. In contrast, in our study, we found initial lesions in the liver of two animals as early as 24 hpi (TP1), including the one animal showing type III lesions in the lung and splenic lesions at this time point. Similar changes in the liver and spleen were observed by Barnes *et al*. (2001), with an early neutrophil influx to the organs causing small inflammatory foci at 24 hpi. Furthermore, we observed an exponential increase in the number of lesions in both liver and spleen, at 48 hpi which continued at 60 hpi although the challenge dose used in the Lever *et al*. (2009) study (100 cfu) was similar to the one used in our study.

Intense immunostaining for the capsular polysaccharide antigen of *B. pseudomallei* was observed within lesions in all tissues. The extension and intensity of the staining were higher in large lesions, which could reflect the sustained bacterial growth at these sites.

After analysing the cell composition of microscopic lesions by immunohistochemistry, we have found neutrophils (Ly‐6G+ cells) as the most frequent cells. In a recent comparative virulence study (Massey *et al*. 2014), an important increase (around 300%) in the number of neutrophils in peripheral blood was observed in infected animals. Furthermore, previous studies have demonstrated that activated neutrophils are rapidly recruited to the lungs after a high‐dose pulmonary challenge with *B*. *pseudomallei* using flow cytometry (Easton *et al*. 2007) or histopathology (Barnes *et al*. 2001; Kager *et al*. 2014). This has also been described in another study highlighting the presence of macrophages and natural killer cells in the lesions (Bearss *et al*. 2017). In line with this finding, histological analysis of tissue from human patients with melioidosis has demonstrated the presence of abundant neutrophils together with a small number of macrophages within lesions (Wong *et al*. 1995).

It has been suggested that neutrophils play a role in early resistance against *B. pseudomallei* infection (Breitbach *et al*. 2006). However, our findings are more in agreement with those of the authors who have suggested that neutrophils are not sufficient to control the infection despite their recruitment to the infection site (Massey *et al*. 2014). In addition, Massey *et al*. (2014) found an important increase in IL‐17 in infected animals compared with non‐infected control animals. This cytokine is recognized as the main mechanism whereby Th17 cells contribute to tissue damage due to prolonged neutrophil recruitment (Fogli *et al*. 2013). This mechanism may also be involved in the development of microscopic lesions observed in our study.

There is growing literature supporting the view that an excessive immune response, leading to exaggerated cellular recruitment, is a major contributor to morbidity and ultimately mortality in a number of different infections (D′elia *et al*. 2013). Furthermore, it has been shown that dampening down the immune response during *Burkholderia* spp*.,* infection can increase survival. Inhibition of IL‐1beta, COX‐2 and HMGB1 has been shown to be beneficial in models of melioidosis (Ceballos‐Olvera *et al*. 2011; Asakrah *et al*. 2013; Charoensup *et al*. 2014; Laws *et al*. 2015).

It has also been demonstrated that robust T‐cell functions are paramount in protection against *B. pseudomallei* infection and that IFN‐g and the IFN‐g‐inducing cytokines IL‐12 and IL‐18 are essential for initial resistance (Haque *et al*. 2006). In our study, the number of CD3 +  cells within the lesions was quite low. We found a statistically significant higher presence of CD3 +  cells within early lesions at 24 hpi, which could represent a first attempt to control the disease. However, the presence of CD3 +  cells was minimal at 48 hpi and 60 hpi, where lesions were mainly formed by neutrophils and some macrophages. This could be one of the reasons why BALB/c mice fail to control the disease and die in <5 days after infection (Lever *et al*. 2009). Indeed, the importance of T cells later during infection has been shown in a *B. mallei* model (Rowland *et al*. 2010).

A recent study showed that strong CD4 +  and CD8 +  T‐cell responses are required for patients to survive acute melioidosis (Jenjaroen *et al*. 2015). CD4 +  T cells were also reported to protect mouse in the late stage of *B. pseudomallei* infection (Haque *et al*. 2006). We found a slight increase (not statistically significant) in the presence of CD3 +  cells in type I lesions at TP3 that would not seem to be enough to establish a late response.

B lymphocytes were not observed in any type of lesions in our study independent of the time point analysed, although occasional aggregates of B cells were observed within healthy lung tissue adjacent to lesions. This may be due to the fact that the lesional pattern is too acute for the intervention of this cell population.

This finding has been described in other diseases caused by intracellular pathogens such as *Mycobacterium bovis* (Salguero *et al*. 2016). However, the role of these ‘nests’ in the host immune response is not well understood.

The histopathological and immunohistochemical analysis of the lesions found in this BALB/c mice model demonstrates the high susceptibility of this mouse strain to acute melioidosis. This is corroborated by the highest presence of *B. pseudomallei* antigen within the largest lesions, which are deficient in a T‐cell response and dominated by the presence of neutrophils. The high recruitment of neutrophils seems to be responsible for most of the tissue damage observed within histopathological lesions.

## Sources of funding

The study was funded by United Kingdom Ministry of Defense. The digital pathology workstation used in this study was funded by vHive (Veterinary Health Innovation Engine) at the School of Veterinary Medicine, University of Surrey. W.L. García‐Jiménez acknowledges the Junta de Extremadura and the European Social Fund for his research contract (PO14022).

## Conflict of interest

Authors do not declare any conflict of interest.

## Author contributions

WLG, FJS and RVD equally contributed to the writing of the manuscript. RVD was responsible for the experimental design and performing the animal studies. WLG and FJS were responsible for the histopathological and immunohistochemical characterization.

## References

[iep12258-bib-0001] Aldhous P. (2005) Melioidosis? Never heard of it. Nature 434, 692–693. 15815599 10.1038/434692a

[iep12258-bib-0002] Asakrah S. , Nieves W. , Mahdi Z. *et al* (2013) Post‐Exposure Therapeutic Efficacy of COX‐2 Inhibition against *Burkholderia pseudomallei* . PLoS Negl. Trop Dis. 7, e2212. 23675544 10.1371/journal.pntd.0002212 PMC3649956

[iep12258-bib-0003] Barnes J.L. & Ketheesan N. (2005) Route of infection in melioidosis [5]. Emerging Infect. Dis. 11, 638–639. 15834987 10.3201/eid1104.041051 PMC3320332

[iep12258-bib-0004] Barnes J.L. , Ulett G.C. , Ketheesan N. , Clair T. , Summers P.M. & Hirst R.G. (2001) Induction of multiple chemokine and colony‐stimulating genes in experimental *Burkholderia pseudommalei* infection. Immunol. Cell Biol. 79, 490–501. 11564157 10.1046/j.1440-1711.2001.01038.x

[iep12258-bib-0005] Bearss J.J. , Hunter M. , Dankmeyer J.L. *et al* (2017) Characterization of pathogenesis of and immune response to *Burkholderia pseudomallei* K96243 using both inhalational and intraperitoneal infection models in BALB/c and C57BL/6 mice. PLoS One 12, e0172627. 28235018 10.1371/journal.pone.0172627 PMC5325312

[iep12258-bib-0006] Breitbach K. , Klocke S. , Tschernig T. *et al* (2006) Role of inducible nitric oxide synthase and NADPH oxidase in early control of *Burkholderia pseudomallei* infection in mice. Infect. Immun. 74, 6300–6309. 17000727 10.1128/IAI.00966-06 PMC1695503

[iep12258-bib-0007] Ceballos‐Olvera I. , Sahoo M. , Miller M.A. , del Barrio L. & Re F. (2011) Inflammasome‐dependent pyroptosis and IL‐18 protect against *Burkholderia pseudomallei* lung infection while IL‐1B is deleterious. PLoS Pathog. 7, e1002452. 22241982 10.1371/journal.ppat.1002452 PMC3248555

[iep12258-bib-0008] Charoensup J. , Sermswan R.W. , Paeyao A. *et al* (2014) High HMGB1 level is associated with poor outcome of septicemic melioidosis. Int. J. Infec. Dis. 28, e111–e116. 10.1016/j.ijid.2014.07.025 25263503

[iep12258-bib-0009] Conejero L. , Patel N. , De Reynal M. *et al* (2011) Low‐dose exposure of C57BL/6 mice to *Burkholderia pseudomallei* mimics chronic human melioidosis. Am. J. Path. 179, 270–280. 21703409 10.1016/j.ajpath.2011.03.031 PMC3123849

[iep12258-bib-0010] D′elia R.V. , Harrison K. , Oyston P.C. , Lukaszewski R.A. , Clark G.C. (2013) Targeting the “Cytokine Storm” for Therapeutic Benefit. Clin. Vac. Immunol. 20, 319–327. 10.1128/CVI.00636-12 PMC3592351 23283640

[iep12258-bib-0011] Dance D. (2014) Treatment and prophylaxis of melioidosis. Int. J. Antimicrob. Agents 43, 310–318. 24613038 10.1016/j.ijantimicag.2014.01.005 PMC4236584

[iep12258-bib-0012] David J. , Bell R.E. & Clark G.C. (2015) Mechanisms of disease: host‐pathogen interactions between burkholderia species and lung epithelial cells. Front. Cel. Infect. Microbiol. 5 DOI: https://www.frontiersin.org/articles/10.3389/fcimb.2015.00080/full 10.3389/fcimb.2015.00080 PMC4649042 26636042

[iep12258-bib-0013] Druett H.A. (1969) A mobile form of the Henderson apparatus. J. Hyg. 67, 437–448. 5258223 10.1017/s0022172400041851 PMC2130736

[iep12258-bib-0014] Easton A. , Haque A. , Chu K. , Lukaszewski R. & Bancroft G.J. (2007) A critical role for neutrophils in resistance to experimental infection with *Burkholderia pseudomallei* . J. Infect. Dis. 195, 99–107. 17152013 10.1086/509810

[iep12258-bib-0015] Fogli L.K. , Sundrud M.S. , Goel S. *et al* (2013) T cell‐derived IL‐17 mediates epithelial changes in the airway and drives pulmonary neutrophilia. J. Immunol. 191, 3100–3111. 23966625 10.4049/jimmunol.1301360 PMC3822005

[iep12258-bib-0016] Gilad J. , Harary I. , Dushnitsky T. , Schwartz D. & Amsalem Y. (2007) *Burkholderia mallei* and *Burkholderia pseudomallei* as bioterrorism agents: national aspects of emergency preparedness. Isr. Med. Assoc. J. 9, 499–503. 17710778

[iep12258-bib-0017] Haque A. , Easton A. , Smith D. *et al* (2006) Role of T cells in innate and adaptive immunity against murine *Burkholderia pseudomallei* infection. J. Infect. Dis. 193, 370–379. 16388484 10.1086/498983

[iep12258-bib-0018] Hoppe I. , Brenneke B. , Rohde M. *et al* (1999) Characterization of a murine model of melioidosis: comparison of different strains of mice. Infect. Immun. 67, 2891–2900. 10338496 10.1128/iai.67.6.2891-2900.1999 PMC96597

[iep12258-bib-0019] Jeddeloh J.A. , Fritz D.L. , Waag D.M. , Hartings J.M. & Andrews G.P. (2003) Biodefense‐driven murine model of pneumonic melioidosis. Infect. Immun. 71, 584–587. 12496217 10.1128/IAI.71.1.584-587.2003 PMC143420

[iep12258-bib-0020] Jenjaroen K. , Chumseng S. , Sumonwiriya M. *et al* (2015) T‐Cell responses are associated with survival in acute melioidosis patients. PLoS Negl. Trop Dis. 9, e0004152. 26495852 10.1371/journal.pntd.0004152 PMC4619742

[iep12258-bib-0021] Kaestli M. , Mayo M. , Harrington G. *et al* (2009) Landscape changes influence the occurrence of the melioidosis bacterium *Burkholderia pseudomallei* in soil in Northern Australia. PLoS Negl. Trop Dis. 3, e364. 19156200 10.1371/journal.pntd.0000364 PMC2617783

[iep12258-bib-0022] Kager L.M. , Wiersinga W.J. , Roelofs J.T.H. *et al* (2014) A thrombomodulin mutation that impairs active protein C generation is detrimental in severe pneumonia‐derived gram‐negative sepsis (melioidosis). PLoS Negl. Trop Dis. 8, e2819. 24762740 10.1371/journal.pntd.0002819 PMC3998929

[iep12258-bib-0023] Laws T.R. , Clark G.C. & D'Elia R.V. (2015) Immune profiling of the progression of a BALB/c mouse aerosol infection by *Burkholderia pseudomallei* and the therapeutic implications of targeting HMGB1. Int. J. Infec. Dis. 40, 1–8. 26358857 10.1016/j.ijid.2015.09.003

[iep12258-bib-0024] Leakey A.K. , Ulett G.C. & Hirst R.G. (1998) BALB/c and C57BI/6 mice infected with virulent *Burkholderia pseudomallei* provide contrasting animal models for the acute and chronic forms of human melioidosis. Microb. Pathog. 24, 269–275. 9600859 10.1006/mpat.1997.0179

[iep12258-bib-0025] Leelarasamee A. (1998) *Burkholderia pseudomallei*: the unbeatable foe? Southeast Asian J. Trop. Med. Public Health 29, 410–415. 9886137

[iep12258-bib-0026] Lever M.S. , Nelson M. , Stagg A.J. , Beedham R.J. & Simpson A.J.H. (2009) Experimental acute respiratory *Burkholderia pseudomallei* infection in BALB/c mice. Int. J. Exper. Dis. 90, 16–25. 10.1111/j.1365-2613.2008.00619.x PMC2669612 19200247

[iep12258-bib-0027] Liu B. , Koo G.C. , Yap E.H. , Chua K.L. & Gan Y.H. (2002) Model of differential susceptibility to mucosal *Burkholderia pseudomallei* infection. Infect. Immun. 70, 504–511. 11796576 10.1128/IAI.70.2.504-511.2002 PMC127661

[iep12258-bib-0028] Massey S. , Yeager L.A. , Blumentritt C.A. *et al* (2014) Comparative *Burkholderia pseudomallei* natural history virulence studies using an aerosol murine model of infection. Sci. Rep. 4, 4305. 24603493 10.1038/srep04305 PMC3945929

[iep12258-bib-0029] May K.R. & Harper G.J. (1957) The efficiency of various liquid impinger samplers in bacterial aerosols. Br. J. Ind. Med. 14, 287–297. 13471876 10.1136/oem.14.4.287 PMC1037828

[iep12258-bib-0030] Mima T. & Schweizer H.P. (2010) The BpeAB‐OprB efflux pump of *Burkholderia pseudomallei* 1026b does not play a role in quorum sensing, virulence factor production, or extrusion of aminoglycosides but is a broad‐spectrum drug efflux system. Antimicrob. Agents Chemother. 54, 3113–3120. 20498323 10.1128/AAC.01803-09 PMC2916348

[iep12258-bib-0031] Rhodes K.A. & Schweizer H.P. (2016) Antibiotic resistance in Burkholderia species. Drug. Resist. Updat. 28, 82–90. 27620956 10.1016/j.drup.2016.07.003 PMC5022785

[iep12258-bib-0032] Rowland C.A. , Lever M.S. , Griffin K.F. , Bancroft G.J. & Lukaszewski R.A. (2010) Protective cellular responses to Burkholderia mallei infection. Microbes Infect. 12, 846–853. 20542133 10.1016/j.micinf.2010.05.012

[iep12258-bib-0033] Salguero F.J. , Gibson S. , Garcia‐Jimenez W. *et al* (2016) Differential cell composition and cytokine expression within lymph node granulomas from BCG‐vaccinated and non‐vaccinated Cattle Experimentally Infected with *Mycobacterium bovis* . Transb. Emerg. Dis. 64, 1734 In press https://doi.org/10.1111/tbed.12561 10.1111/tbed.12561 27615603

[iep12258-bib-0034] Srinivasan A. , Kraus C. , Deshazer D. *et al* (2001) Glanders in a military research microbiologist. New Engl. J. Med. 345, 256–258. 11474663 10.1056/NEJM200107263450404

[iep12258-bib-0035] Tan G.Y.G. , Liu Y. , Sivalingam S.P. *et al* (2008) *Burkholderia pseudomallei* aerosol infection results in differential inflammatory responses in BALB/c and C57Bl/6 mice. J. Med. Microbiol. 57, 508–515. 18349373 10.1099/jmm.0.47596-0

[iep12258-bib-0036] Ulett G.C. , Ketheesan N. & Hirst R.G. (2000) Cytokine gene expression in innately susceptible BALB/c mice and relatively resistant C57BL/6 mice during infection with virulent *Burkholderia pseudomallei* . Infect. Immun. 68, 2034–2042. 10722599 10.1128/iai.68.4.2034-2042.2000 PMC97383

[iep12258-bib-0037] Valade E. , Thibault F.M. , Biot F.V. & Vidal D.R. (2009) Melioidosis: an emerging tropical disease. Med. Trop. 69, 437–445. 20025169

[iep12258-bib-0038] Wiersinga W.J. , van der Poll T. , White N.L. , Day N.P. & Peacock S.J. (2006) Melioidosis: insights into the pathogenicity of *Burkholderia pseudomallei* . Nat. Rev. Microbiol. 4, 272–282. 16541135 10.1038/nrmicro1385

[iep12258-bib-0039] Wiersinga W.J. , Veer C.V. , Van Den Pangaart P.S. *et al* (2009) Immunosuppression associated with interleukin‐1R‐associated‐kinase‐M upregulation predicts mortality in Gram‐negative sepsis (melioidosis). Crit. Care Med. 37, 569–576. 19114913 10.1097/CCM.0b013e318194b1bf

[iep12258-bib-0040] Wong K.T. , Puthucheary S.D. & Vadivelu J. (1995) The histopathology of human melioidosis. Histopathology 26, 51–55. 7713483 10.1111/j.1365-2559.1995.tb00620.x

